# Design and Study of a PVDF Piezoelectric Film Force Sensor Based on Interface Force Field Reconstruction and Surface Domain Segmentation

**DOI:** 10.3390/mi17020262

**Published:** 2026-02-19

**Authors:** Kaiqiang Yan, Wenge Wu, Xinyi Wu, Yunping Cheng, Lijuan Liu, Yongjuan Zhao, Yicheng Zhang, Pengcheng Liu, Zhi Wang

**Affiliations:** 1 School of Mechanical Engineering, North University of China, Taiyuan 030051, China; b20230205@st.nuc.edu.cn (K.Y.); ypchengbk@163.com (Y.C.); liulijuan@nuc.edu.cn (L.L.); zyj@nuc.edu.cn (Y.Z.); 16622890030@163.com (Y.Z.); sz202302037@st.nuc.edu.cn (P.L.); 18735887532@163.com (Z.W.); 2 School of Automation Engineering, University of Electronic Science and Technology of China, Chengdu 611731, China; wxywuxinyi@sina.com

**Keywords:** PVDF piezoelectric film, force sensor, interface force field reconstruction, surface domain segmentation, three-dimensional force

## Abstract

The accurate measurement of dynamic forces is pivotal for advancing manufacturing process monitoring and enhancing equipment intelligence. To address the challenges of contact interface force field nonlinearity in existing PVDF piezoelectric film force sensors and the inability of a monolithic PVDF piezoelectric film to measure multi-dimensional forces, this study designs a uniform-load double-bossed elastic force-transmitting diaphragm to achieve contact interface force field reconstruction between the sensor’s elastic sensing structure and the sensitive element group. Building upon the load-bearing surface domain segmentation technique, the silver ink electrode on the front surface of a complete circular PVDF piezoelectric film is segmented into four independent sector-shaped rings. Each sector ring, together with its underlying PVDF piezoelectric film, constitutes a sensitive element, and these four sensitive elements are integrated to form the sensitive element group. The three-dimensional force measurement method of this sensitive element group in the Cartesian coordinate system is investigated. The measurement of three-dimensional force is realized by leveraging the tensile-compressive piezoelectric effect of each sensitive element in conjunction with a pre-stressed assembly structure. Quasi-static calibration test results indicate that the charge sensitivities of the force sensor in the X-, Y-, and Z-directions are 52.63 pC/N, 55.96 pC/N, and 9.02 pC/N, respectively, with a linearity ≤4.6%. Dynamic calibration test results reveal that the force measurement module exhibits a natural frequency of 4675.5 Hz. Experimental investigations into the response of triaxial cutting forces to variations in cutting speed, feed rate, and cutting depth were conducted, which verified the sensor’s ability to capture dynamic three-dimensional cutting forces. This study provides an effective solution for the structural design and three-dimensional force measurement methodology of PVDF piezoelectric film force sensors.

## 1. Introduction

Dynamic force measurement serves as a fundamental technical cornerstone for modern smart manufacturing, robotics, and condition monitoring of high-end equipment. Its accuracy and reliability directly impact process optimization, quality control, and the level of equipment intelligence [1,2,3,4]. Among various force measurement techniques, piezoelectric force sensors are particularly suitable for real-time monitoring of dynamic and transient forces due to their high-frequency response, high stiffness, and excellent dynamic performance [5,6,7,8,9,10,11,12]. The polyvinylidene fluoride (PVDF) piezoelectric film, characterized by its high flexibility, wide frequency response, and ease of shaping, offers an ideal material choice for developing dynamic force sensors [13,14,15,16,17,18,19,20,21].

In recent years, research on PVDF piezoelectric film force sensors has primarily focused on two key aspects: structural design and film arrangement. Nguyen et al. [22,23] directly attached PVDF piezoelectric films to the surface of a tool shank, utilizing their *d*_31_ tensile mode to sense the bending or torsional strain induced by cutting forces. Liu et al. [24] placed two PVDF piezoelectric films between clamping components in a preloaded configuration, indirectly measuring cutting forces through the dynamic pressure exerted on the films. Li et al. [25,26] designed a sensor structure consisting of a lower cover, an upper cover, and a PVDF piezoelectric film for dynamic force monitoring during cold forging processes. Luo et al. [27] embedded three PVDF piezoelectric films at non-orthogonal angles into the bottom and sides of a tool holder. A decoupling model based on spatial force equilibrium was established, enabling wireless measurement of three-dimensional cutting forces. The same research team [28] further designed a benchtop milling force dynamometer, arranging a total of seven PVDF piezoelectric films on three positioning surfaces and the bottom surface of an inner worktable. This spatial arrangement enabled the measurement of force components in various directions. Wang et al. [29] embedded a three-layer stack of PVDF piezoelectric films into the sensor housing, enabling three-dimensional force perception through the charge outputs from different film layers. In a subsequent study [30], the team measured tangential forces using a diamond-symmetric column structure and detected normal forces with PVDF piezoelectric films placed between the upper and lower cover plates. This configuration exhibits the capability to mitigate inter-axis coupling. G. Totis et al. [31] arranged eight PVDF piezoelectric films within a rigid structure. By employing a four-beam force transmission mechanism, the films were primarily subjected to normal pressure, which significantly reduced shear interference. The reliability of this design was validated through milling experiments. Yu et al. [32] proposed a flexible piezoelectric tactile sensor array for three-axis dynamic force measurement. The three-dimensional contact force measurement was achieved through the structural coordination of four piezoelectric capacitors and differential signal processing. Ting et al. [33] innovatively integrated six patterned electrodes on a PVDF piezoelectric film to form three electrode pairs, which were used to detect the bending and torsional deformations of the tool induced by cutting forces.

However, existing research has primarily focused on the arrangement of sensing elements and signal decoupling methods. Multi-axis force measurement typically relies on the spatial distribution of multiple independent PVDF piezoelectric films. While this approach achieves multi-dimensional force sensing, it often introduces more assembly interfaces, more complex signal wiring, higher risks of unit inconsistency, and increases the overall sensor size and manufacturing cost. Furthermore, there is still a lack of systematic structural innovation starting from the force field at the contact interface between the sensor housing and the sensing elements. To address this gap, this study proposes a novel design approach based on the synergistic integration of interface force field reconstruction and surface domain segmentation using a monolithic circular PVDF piezoelectric film. This method enables three-dimensional force measurement through an integrated single sensing element, fundamentally reducing the number of sensing units and assembly layers while enhancing sensor consistency, compactness, and cost-effectiveness.

## 2. Structural Design and Measurement Principle of the PVDF Piezoelectric Film Force Sensor

### 2.1. Design of a PVDF Piezoelectric Film Force Sensor Based on Interface Force Field Reconstruction

**Figure 1:**
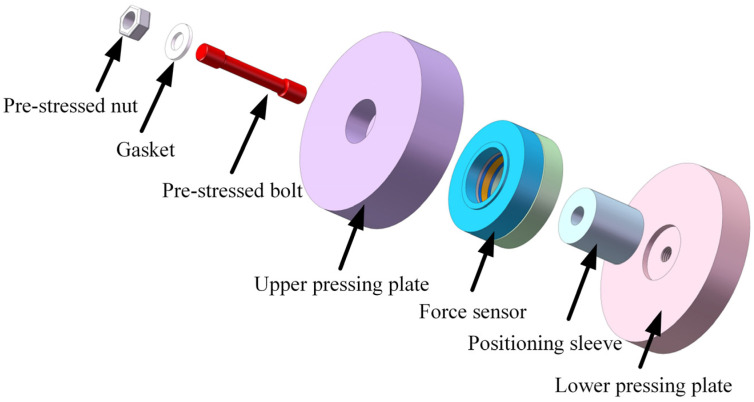
Schematic diagram of the overall assembly structure of the three-dimensional PVDF piezoelectric film force measurement module.

The overall assembly structure of the three-dimensional force measurement module with a PVDF piezoelectric film, designed in this study, is shown in Figure 1. Its core component is the PVDF piezoelectric film force sensor, which is integrated via a pre-stressed assembly structure. This structure ensures the stability of the overall assembly while providing the sensor with an ideal linear operating range.

The measurement process of the PVDF piezoelectric film force sensor is essentially a chain transmission of force-to-electricity conversion. During the sensor measurement process, the PVDF piezoelectric film converts mechanical energy into electrical energy based on the direct piezoelectric effect, and its piezoelectric constitutive relation is given by Equation (1). To ensure that the internally generated charge density maintains a favorable linear response under an external linear load, the following conditions must be satisfied: first, the stress distribution on the PVDF piezoelectric film must be uniform; second, the effective load-bearing area of the PVDF piezoelectric film that actually participates in the force-to-electricity conversion must remain constant; and finally, its operating mode should be dominated as much as possible by the thickness-direction piezoelectric coefficient *d*_33_, with minimal composite contribution from the *d*_31_/*d*_32_ modes.

(1) $$Q=d_{33}\cdot F_{b}=d_{33}\cdot \sigma \cdot A$$

In the equation, *Q* is the charge generated on the surface of the PVDF piezoelectric film, *d*_33_ is the piezoelectric constant of the PVDF film, *F_b_* is the force acting on the PVDF piezoelectric film, σ is the stress acting on the PVDF piezoelectric film, and *A* is the area over which the force acts.

**Figure 2:**
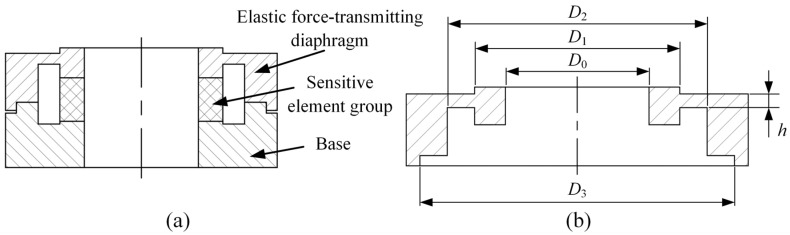
Cross-sectional views of the sensor structure and the uniform-load double-bossed elastic force-transmitting diaphragm. (**a**) Cross-sectional view of the sensor and (**b**) structure of the uniform-load double-bossed elastic force-transmitting diaphragm.

Figure 2a is a cross-sectional view of the sensor, which consists of three parts: the elastic force-transmitting diaphragm 1, the PVDF piezoelectric film sensitive element group 2, and the base 3. The bosses on both the elastic force-transmitting diaphragm and the base jointly clamp the PVDF piezoelectric film sensitive element group. The elastic force-transmitting diaphragm serves as the key component that directly receives the external load and transmits it to the PVDF piezoelectric film sensitive element group. Its mechanical characteristics have a decisive impact on the sensor’s performance. The key to achieving high-precision force-to-electricity conversion lies in providing the PVDF piezoelectric film sensitive element group with an ideal and stable mechanical contact interface.

To this end, this study designs a uniform-load double-bossed elastic force-transmitting diaphragm to reconstruct and optimize the contact interface force field between the elastic force-transmitting diaphragm and the PVDF piezoelectric film sensitive element group. Its structure is shown in Figure 2b. The main structural features of this elastic force-transmitting diaphragm include a central through-hole, upper and lower double bosses, an elastic ring, and a boundary support. The relevant geometric parameters are defined as follows: the through-hole radius is *R*_0_, the upper and lower boss radii are *R*_1_, the elastic ring radius is *R*_2_, the elastic ring thickness is *h*, and the boundary support radius is *R*_3_.

Due to the high overall stiffness of the PVDF piezoelectric film force sensor, its deformation under load is minimal. Within the sensor’s designed measurement range, all components operate in the elastic regime. This study focuses on the elastic force-transmitting diaphragm as the core component, investigating its mechanical behavior under load to reveal the underlying mechanism of interface force field reconstruction. Accordingly, the following assumptions are adopted in the theoretical modeling of the standalone elastic force-transmitting diaphragm: the ratio of the maximum deflection of the uniform-load double-bossed elastic force-transmitting diaphragm to the thickness of its elastic ring is less than 0.5, thus small-deflection theory is applicable. The load *F* is uniformly applied to the surface of the upper boss. The stiffness of the upper and lower bosses is considerably higher than that of the elastic ring; therefore, they are modeled as rigid bodies. Accordingly, only the deformation of the elastic ring is considered, and the bosses themselves undergo no deformation. Under actual assembly conditions, the sensor exhibits two parallel load transmission paths: one path acts on the PVDF piezoelectric film sensitive element group via the lower boss and continues downward to the base; the other path is transmitted downward to the base sequentially through the elastic ring and the boundary support. Under this parallel load transmission path, the mutual compression between the lower boss and the sensitive element group induces local deformation of the boss, whereas when the standalone elastic force-transmitting diaphragm is loaded alone, the boss itself undergoes no such deformation. Therefore, although the theoretical assumptions in this section do not cover all load transmission paths of the sensor, they do not affect the elucidation of the mechanical mechanism of the core component. Based on the theory of elasticity, the expression for the deflection ω(r) of the elastic ring of the uniform-load double-bossed elastic force-transmitting diaphragm is derived, as shown in Equation (2).

(2) $$\omega (r)=\frac{3F(1-\mu^{2})R_{2}^{2}}{4\pi Eh^{3}}\left[G^{2}(2\ln G-B-1)+2B\ln G+B+1\right]$$

In the equations, $G=\frac{r}{R_{2}}$, $K=\frac{R_{1}}{R_{2}}$, $B=-\frac{2K^{2}\ln K}{1-K^{2}}$.

Based on the axisymmetric bending theory of circular plates, the expressions for the radial stress $\sigma_{r}$ and tangential stress $\sigma_{t}$ on the lower surface of the elastic ring in the uniform-load double-bossed elastic force-transmitting diaphragm are further derived, as given in Equation (3).

(3) $$\left\{\begin{array}{c} \sigma_{r}=-\frac{3F}{4\pi h^{2}}\left[\left(2\ln G+2-B-\frac{B}{G^{2}})+\mu (2\ln G-B+\frac{B}{G^{2}}\right)\right]\\ \sigma_{t}=-\frac{3F}{4\pi h^{2}}\left[\left(2\ln G-B+\frac{B}{G^{2}})+\mu (2\ln G+2-B-\frac{B}{G^{2}}\right)\right] \end{array}\right.$$

According to the generalized Hooke’s law, the expressions for the radial strain $\varepsilon_{r}$ and tangential strain $\varepsilon_{t}$ on the lower surface of the elastic ring in the uniform-load double-bossed elastic force-transmitting diaphragm are further derived, as shown in Equation (4).

(4) $$\left\{\begin{array}{l} \varepsilon_{r}=-\frac{3F(1-\mu^{2})}{4\pi Eh^{2}}(2\ln G+2-B-\frac{B}{G^{2}})\\ \varepsilon_{t}=-\frac{3F(1-\mu^{2})}{4\pi Eh^{2}}(2\ln G-B+\frac{B}{G^{2}}) \end{array}\right.$$

**Figure 3:**
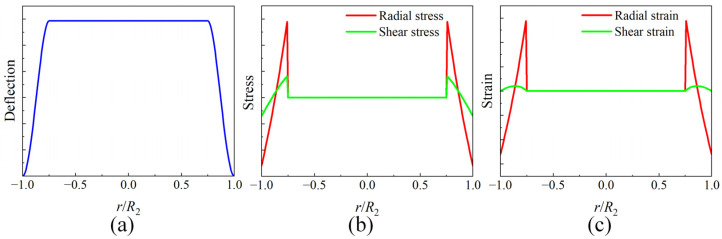
Distribution curves of the mechanical properties for the loaded uniform-load double-bossed elastic force-transmitting diaphragm. (**a**) Deflection distribution curve; (**b**) stress distribution curve; and (**c**) strain distribution curve.

Based on the above analytical model, with the material’s Poisson’s ratio set as μ=0.27 and the structural coefficient as K=0.75. Numerical simulation was then performed using MATLAB R2024b to obtain the distribution curves of the mechanical characteristics of the uniform-load double-bossed elastic force-transmitting diaphragm under load, as shown in Figure 3.

The simulation results indicate that the lower surface of the contact interface between this elastic force-transmitting diaphragm and the PVDF piezoelectric film sensitive element group exhibits uniform and stable distributions of deflection, stress, and strain. Simultaneously, mechanical discontinuities are effectively transferred to the elastic ring region, which is not in contact with the sensitive element group. This design creates an ideal mechanical environment for the sensitive element group, characterized by uniform loading, a constant effective loaded area, and a single operating mode, thereby significantly enhancing the consistency and reliability of the sensor’s measurements.

### 2.2. Finite Element Validation and Quantitative Analysis of Sensor Interface Force Field Reconstruction

**Figure 4:**
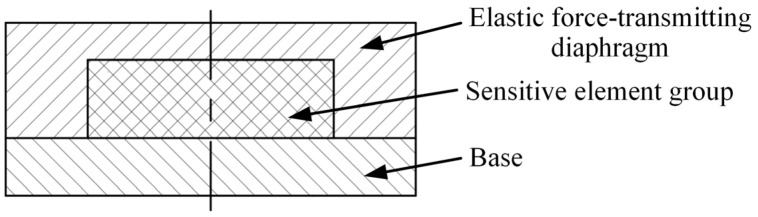
The sensor structure incorporating the circular flat elastic force-transmitting diaphragm.

To quantitatively evaluate the effectiveness of the uniform-load double-bossed elastic force-transmitting diaphragm in contact interface force field reconstruction, this study employs finite element analysis using ANSYS Workbench 2020 R2 to comparatively analyze the mechanical response of the sensor under the uniform-load structure and the conventional circular flat structure. The sensor structure incorporating the circular flat elastic force-transmitting diaphragm is shown in Figure 4, in which the contact surface with the PVDF piezoelectric film is a single plane.

To ensure consistency in the comparison, the elastic ring thickness of both structures was set to 2 mm and the outer diameter to 38 mm; the outer diameter of the PVDF piezoelectric film was uniformly set to 30 mm. The performance parameters of the PVDF piezoelectric film are listed in Table 1.

The load application method was defined according to the structural characteristics: for the uniform-load structure, the load was applied to the surface of the upper boss; for the circular flat structure, a concentrated load was applied at the center of the diaphragm. Under the same axial load (*F* = 2000 N), the lower surface of the base was set as a fixed support. A bonded contact was defined between the elastic force-transmitting diaphragm and the base, while the remaining contact interfaces were modeled as frictional contacts with a friction coefficient of 0.3. The uniform-load structure model consisted of 59,989 nodes and 30,209 elements, and the circular flat structure model comprised 61,711 nodes and 11,136 elements. The material properties of each sensor component are listed in Table 2.

**Figure 5:**
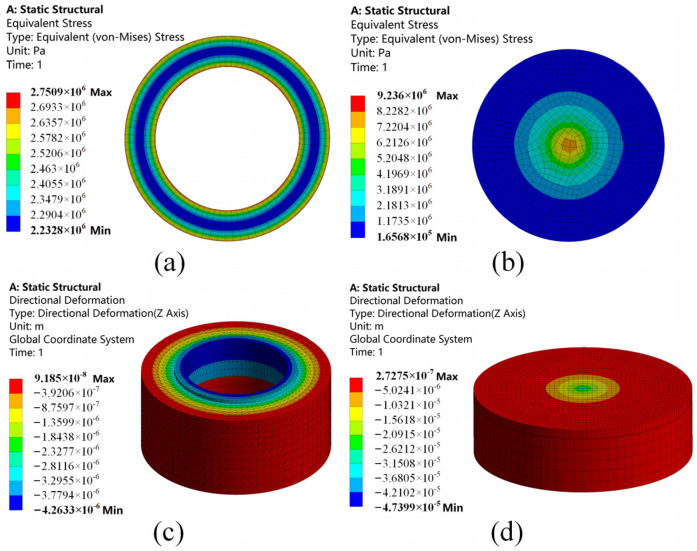
Equivalent stress contour of the PVDF piezoelectric film and overall deformation contour of the sensor under the two sensor structures. (**a**) Equivalent stress distribution contour of the piezoelectric film under the uniform-load structure; (**b**) equivalent stress contour of the piezoelectric film under the circular flat structure; (**c**) overall deformation contour of the sensor under the uniform-load structure; and (**d**) overall deformation contour of the sensor under the circular flat structure.

Figure 5 presents the equivalent stress distribution of the PVDF piezoelectric film and the deformation contour of the sensor under both structures. To objectively evaluate the uniformity of the stress distribution, the stress concentration factor *K_t_* and the dimensionless displacement ratio *W* were defined as key indicators. Their expressions are as follows:

(5) $$\left\{\begin{array}{c} K_{t}=\frac{\sigma_{\max}}{\sigma_{avg}}\\ W=\frac{\omega_{\max}}{h} \end{array}\right.$$

A comparison of the key mechanical indicators between the two structures is presented in Table 3. Analysis reveals that under the circular flat diaphragm structure, the stress concentration factor of the PVDF piezoelectric film reaches as high as 8.19, indicating a highly non-uniform stress distribution with a local stress peak exceeding 9 MPa. This may lead to a nonlinear response in sensor output and even premature localized material failure. In contrast, the stress concentration factor under the uniform-load diaphragm structure is only 1.13, representing an 86.2% reduction compared to the conventional structure, which is close to an ideal uniform stress state (*K_t_* = 1). This result directly verifies that the proposed interface force field reconstruction structure effectively transfers the mechanical discontinuity to the non-contact elastic ring region, thereby providing a highly uniform and stable working stress field for the PVDF piezoelectric film. Meanwhile, the dimensionless displacement ratios of both structures under a 2000 N load are considerably less than 0.5, which validates the assumption of small-deflection theory.

### 2.3. PVDF Piezoelectric Film Sensitive Element Group Based on Surface Domain Segmentation and Its Three-Dimensional Force Measurement Principle

**Figure 6:**
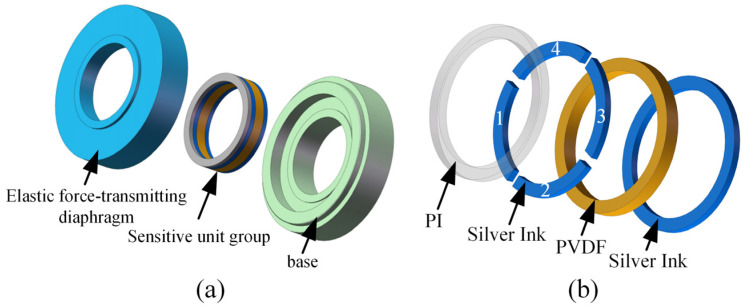
Assembly structure of the sensor and the PVDF piezoelectric film sensitive element group. (**a**) Assembly structure of the sensor. (**b**) Assembly structure of the PVDF piezoelectric film sensitive element group.

The assembly structure of the sensor is shown in Figure 6a. The PVDF piezoelectric film sensitive element group serves as the core sensing element, with its assembly details illustrated in Figure 6b. This sensitive element group features a multilayer structure, consisting of a PI film, a PVDF piezoelectric film, and silver ink electrodes on both sides. Specifically: the first layer is a PI film, which isolates the sensitive element group from the sensor housing to provide electrical insulation; the second layer consists of silver ink electrodes. Based on the load-bearing surface domain segmentation technique, a complete ring of silver ink electrodes is segmented into four independent sector-shaped rings of equal geometric dimensions, labeled as Silver Ink 1–4, each with its own dedicated signal lead-out; the third layer is the PVDF piezoelectric film, which converts the applied mechanical force into a charge signal via the direct piezoelectric effect; the fourth layer is a complete ring of silver ink electrodes, in direct contact with the sensor’s lower cover, grounding the sensitive element group to the sensor housing. Each sector-shaped silver ink ring in the second layer, together with the underlying PVDF piezoelectric film, constitutes one sensitive element. These four sensitive elements constitute the PVDF piezoelectric film sensitive element group.

**Figure 7:**
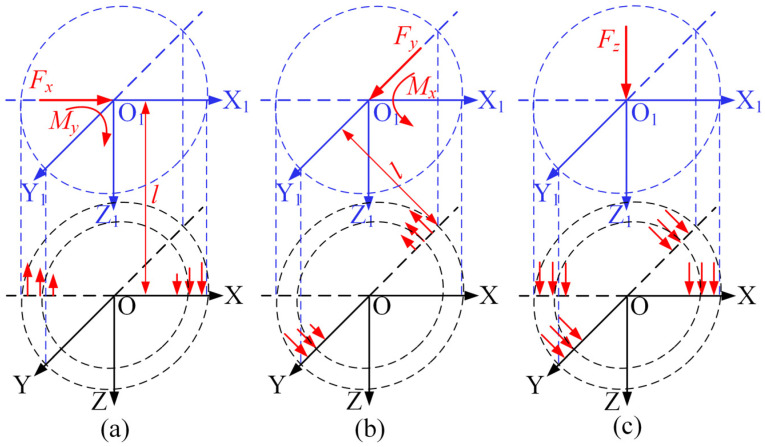
Schematic diagram of the three-dimensional force measurement principle. (**a**) Schematic of *F_x_* measurement principle; (**b**) schematic of *F_y_* measurement principle; and (**c**) schematic of *F_z_* measurement principle.

The schematic diagram illustrating the three-dimensional force measurement principle of the sensor in the Cartesian coordinate system is shown in Figure 7. O_1_-X_1_Y_1_Z_1_ represents the loading coordinate system, and O-XYZ denotes the coordinate system of the PVDF piezoelectric film sensitive element group’s mounting position. The perpendicular distance between the two coordinate systems is *l*. Figure 7a illustrates the measurement principle for the force component *F_x_*. When a positive load *F_x_* is applied in the loading coordinate system, the perpendicular distance *l* between the two coordinate systems acts as a moment arm, subjecting the sensitive element group to a bending moment *M_y_*. Under this condition, the corresponding Silver Ink 2 and 4 experience tension and compression, respectively, generating charge signals *Q*_2_ and *Q*_4_ with opposite polarities. Figure 7b illustrates the measurement principle for the force component *F_y_*. Its principle is consistent with that of *F_x_*. In this direction, Silver Ink 1 and 3 are subjected to tension and compression, respectively, generating charge signals *Q*_1_ and *Q*_3_ with opposite polarities. Figure 7c illustrates the measurement principle for the force component *F_z_*. When *F_z_* is applied in the loading coordinate system, Silver Ink 1, 2, 3, and 4 all bear compressive loads, generating charge signals *Q*_1_, *Q*_2_, *Q*_3_, and *Q*_4_ with identical polarity.

### 2.4. A Collaborative Sensor Design Method Based on Pre-Stressed Assembly Structure and Sensitive Element Group Signal Extraction

Pre-stress is fundamental to enabling bidirectional linear measurement in PVDF piezoelectric film force sensors. Under ideal conditions, the sensitive element group acquires an initial compressive stress $\sigma_{prestress}$ after pre-tightening, and its maximum allowable compressive stress is $\sigma_{\max}$. The measurement range of the sensor is thus determined by the stress interval. During sensor operation, an external load *F* induces an additional stress Δσ on the sensitive element: when *F* is a compressive load, it produces +Δσ; when *F* is a tensile load, it produces −Δσ. To prevent the sensitive element group from detaching or overloading, the condition given in Equation (6) must be satisfied.

(6) $$\left\{\begin{array}{l} \sigma_{prestress}+\Delta \sigma <\sigma_{\max}\\ \sigma_{prestress}-\left|\Delta \sigma \right|>0 \end{array}\right.$$

Therefore, the sensor’s theoretical full-scale range *F_full-scale_*, can be decomposed into two components: the compression range *F_compression_*, corresponding to the interval from the pre-stressed state to the maximum allowable compressive state, and the tension range *F_tension_*, corresponding to the interval from the pre-stressed state to zero stress. Under ideal conditions, neglecting factors such as the stiffness of pre-stress bolts and contact nonlinearities, the relationship among these three quantities is given by Equation (7).

(7) $$\left\{\begin{array}{l} F_{full\;scale}=F_{compression}+F_{tension}\\ F_{tension}\approx F_{prestress} \end{array}\right.$$

In the equation, *F_prestress_* is the pre-stress applied to the sensor.

The pre-stressed assembly structure of the PVDF piezoelectric film force sensor is shown in Figure 1. This structure mainly consists of a pre-stress nut, washers, pre-stress bolt, an upper pressure plate, the force sensor body, a locating sleeve, and a lower pressure plate. The locating sleeve is used to ensure circumferential positioning accuracy between the force sensor and the pre-stressed structure, guaranteeing symmetrical and stable load transmission.

Based on the layout and signal polarity relationships of the four sector-shaped silver ink in the second layer of the PVDF piezoelectric film sensitive element group, the triaxial loads are determined by extracting the corresponding charge signals

Considering the transfer characteristics of the actual system and introducing calibration coefficients, the three-dimensional force values can be computed using Equation (8). However, when the sensor is subjected to multi-directional loads, the charge signals output from each silver ink in the second layer are essentially linear superpositions of the effects from loads in all directions. Therefore, decoupling is required to extract the independent charge components corresponding to the loads in each direction from the composite signals.

(8) $$\left\{\begin{array}{l} F_{x}=a\frac{Q_{4}-Q_{2}}{d_{33}h}\\ F_{y}=b\frac{Q_{3}-Q_{1}}{d_{33}h}\\ F_{z}=c\frac{Q_{1}+Q_{2}+Q_{3}+Q_{4}}{d_{33}} \end{array}\right.$$

In the equations, *Q_i_* is the output charge corresponding to the *i*-th Silver Ink. The coefficients *a*, *b*, and *c* are the triaxial charge sensitivity coefficients obtained from sensor calibration.

## 3. Calibration and Cutting Tests of the PVDF Piezoelectric Film Force Sensor

### 3.1. Establishing the Calibration System for the PVDF Piezoelectric Film Force Sensor

**Figure 8:**
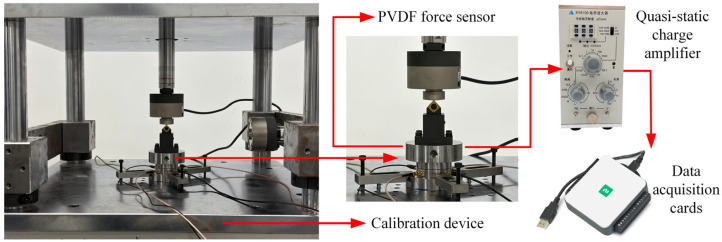
Quasi-static calibration system for the PVDF piezoelectric film force sensor.

To evaluate the static characteristics of the PVDF piezoelectric film force sensor, a quasi-static calibration system was established. As shown in Figure 8, the sensor is mounted on the base plate of the calibration platform, and standard force values are applied via a servo electric cylinder. The charge signals output from the sensor are converted into voltage signals by a quasi-static charge amplifier (XIYUAN XY8100) and then acquired and recorded by a data acquisition card (NI USB-6002). Since dynamic cutting tests will be conducted in Section 3.3, an actual turning tool was installed on the sensor end face during this calibration process to simulate the cutting conditions and perform the calibration accordingly.

During calibration, the high-pass filter of the quasi-static charge amplifier was set to the “L” range, which operates in an approximately DC-coupled state with a lower cut-off frequency of 2 μHz and a time constant greater than 10^5^ s. The loading time for each load step was controlled within 5 s, and the output data were recorded accordingly. Therefore, throughout the entire loading period, signal attenuation caused by charge leakage was negligible, ensuring the reliability of the calibration data.

**Figure 9:**
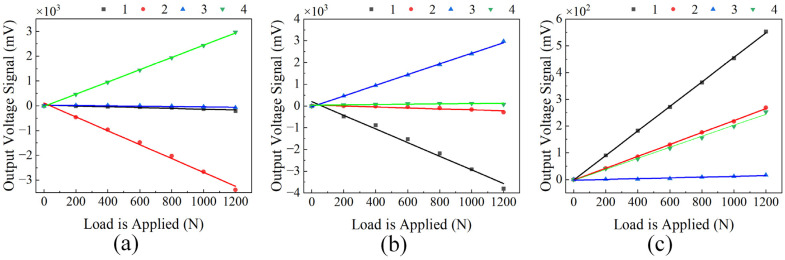
Relationships between the output voltages of the four silver inks and the applied loads under three-directional loading. (**a**) Linear fitting under X-direction loading; (**b**) linear fitting under Y-direction loading; and (**c**) linear fitting under Z-direction loading.

Unidirectional standard loads were applied separately to the sensor in three orthogonal directions, with a load range from 0 N to 1200 N in 200 N increments. The output voltage signals from the sensor were recorded, and linear fitting was performed to obtain the relationships between the output voltages of the four silver ink and the applied loads in the three directions, as shown in Figure 9. Figure 9a,b present the output voltage characteristics of the silver ink under X- and Y-direction loads, respectively. For each lateral load, one of the two corresponding silver ink is subjected to tension while the other is under compression, producing charges of opposite polarity, which aligns with the principle described in Section 2.3. Figure 9c shows the output characteristics of the silver ink under axial loading. Under this load, all four Silver Ink produce charges of the same polarity. However, the output charge levels of Silver Ink 2 and 4 are similar, while those of Silver Ink 1 and 3 are respectively higher and lower. This discrepancy arises because the origin of the loading coordinate system is located at the tool tip, which is not aligned with the sensor’s central axis: when the tool tip is closer to Silver Ink 1, it is simultaneously farther from Silver Ink 3, resulting in a difference in the actual loads experienced by the two Silver Inks.

**Figure 10:**
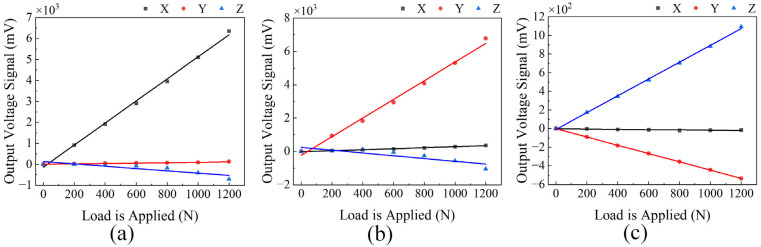
Relationship between the force sensor’s output voltage and the applied load under three-directional loading. (**a**) Linear fitting under X-direction loading; (**b**) Linear fitting under Y-direction loading; (**c**) Linear fitting under Z-direction loading.

Based on the three-dimensional force measurement principle given in Equation (8), the output voltage signals from the four silver inks were processed. This yielded the linear fitting relationships between the sensor’s output voltage and the corresponding applied load in each of the three directions, as shown in Figure 10.

The charge conversion sensitivity of the charge amplifier was set to 10 Nc/V. The relationship between the force sensor’s output voltage and its output charge is given by Equation (9). The main technical specifications of the force sensor are listed in Table 4.

(9) $$Q=V\cdot A_{c}$$

In the equation, *Q* is the output charge of the force sensor, *V* is the output voltage of the charge amplifier, and *A_c_* is the charge conversion sensitivity.

From Table 4, the charge sensitivity of the force sensor in the Z-direction is 9.02 pC/N, while those in the X- and Y-directions are both above 50 pC/N. The Z-direction sensitivity is relatively low, which stems from its measurement mechanism: as shown in Figure 7c, the Z-direction load subjects the four sensing elements to nearly uniform compressive loads, and their charge output is directly governed by the *d*_33_ piezoelectric coefficient of the material, without involving any additional mechanical amplification. In contrast, the X- and Y-direction measurements are based on the lever principle, as illustrated in Figure 7a,b. The transverse load is converted into a bending moment acting on the sensitive element group via the moment arm *l*. This bending moment subjects two opposing sensing elements to tensile and compressive loads, respectively, and the difference between their output charge signals is proportional to the length of the moment arm *l*. Consequently, owing to this mechanical amplification effect, a substantially higher charge sensitivity is achieved compared to that of the Z-direction. In addition, notable cross-axis interference from the X- and Y-directions to the Z-direction exists, primarily due to the offset between the actual loading point (the tool tip) and the origin of the sensor measurement coordinate system. On one hand, the transverse force applied in the X/Y direction generates an additional moment; on the other hand, during loading, the tool tip offset asymmetrically affects the axial preload of the other sensing elements, thereby imposing an additional load on the Z-direction output signal in particular. Linear decoupling can be performed using the least squares method. The input-output relationship can be expressed in matrix form, as shown in Equation (10).

(10) $$F_{s}=G\cdot U$$

In the equation, the matrices *F_s_*, *G*, and *U* represent the applied standard load, the calibration matrix, and the sensor’s output voltage, respectively.

Based on Equation (10), the calibration matrix *G* is obtained using least squares fitting, as shown in Equation (11).

(11) $$G=\left[\begin{array}{ccc} 0.1962 & -0.0098 & 0.0062\\ 0.0047 & 0.1981 & 0.1057\\ 0.0792 & 0.1015 & 1.0176 \end{array}\right]$$

Following the decoupling process, the cross-axis interference errors of the force sensor in all directions were significantly suppressed, as shown in Table 5.

The previously most severe interference errors from the X- and Y-directions to the Z-direction were reduced from 49.55% to 17.49% and from 70.18% to 28.69%, respectively, representing reductions of over 50% in both cases. However, residual cross-axis interference remained after decoupling. This is primarily attributed to the inherent limitations of the least squares-based linear decoupling model adopted in this study. While this method is suitable for decoupling linear or weakly nonlinear systems, certain nonlinear coupling components present in the measurement system could not be fully compensated. These nonlinear couplings mainly arise from two sources: first, nonlinear moment coupling induced by geometric effects caused by the offset of the tool tip; and second, nonlinear coupling interference introduced by minor deviations in the fabrication and assembly of the sensitive element group. Consequently, although the linear decoupling algorithm significantly suppresses the dominant linear coupling components, its compensation capability for the aforementioned nonlinear interferences is limited, resulting in incomplete elimination of the cross-axis interference from the X- and Y-directions to the Z-direction. Nevertheless, the remaining inter-axis interferences were successfully controlled below 2%, demonstrating the effectiveness of the decoupling algorithm and resulting in a notable improvement in the independence and measurement accuracy of each sensor channel.

### 3.2. Dynamic Calibration Test of the PVDF Piezoelectric Film Force Sensor

**Figure 11:**
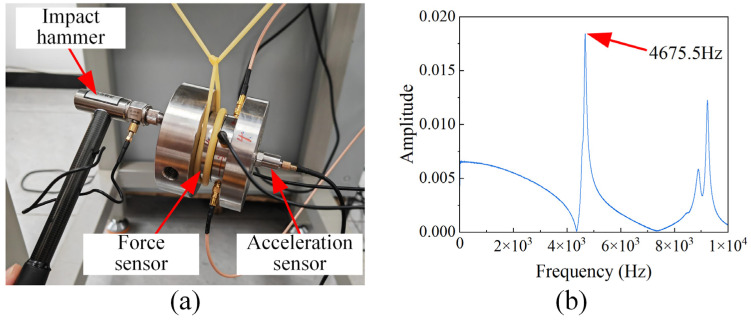
Dynamic calibration test of the sensor and frequency domain characteristic curve. (**a**) Dynamic calibration test of the sensor. (**b**) Frequency domain characteristic curve.

The dynamic performance calibration of the sensor was conducted using an impact test method, with the experimental setup shown in Figure 11a. A free modal test was performed using a natural frequency testing system (Sinocera Piezotronics, Inc., Yangzhou, China, YE6231C). Figure 11b shows that the natural frequency of the force measurement module is 4675.5 Hz.

### 3.3. Cutting Application Validation of the PVDF Piezoelectric Film Force Sensor

**Figure 12:**
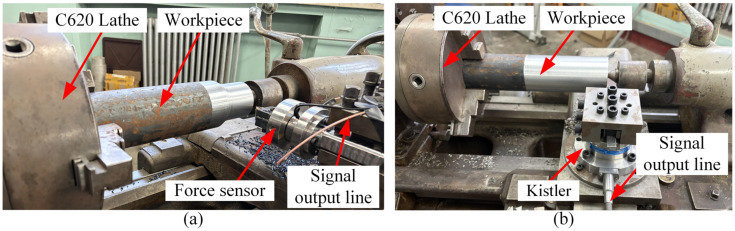
Dynamic cutting test. (**a**) PVDF piezoelectric film force sensor; (**b**) Kistler dynamometer.

To validate the dynamic force measurement performance of the developed sensor, it was integrated into the middle section of the tool structure for dynamic cutting tests. The installation schematic is shown in Figure 12a. The experiments were performed on a variable-speed C620 lathe, using a workpiece made of 45# steel with a diameter of 80 mm. The specific cutting parameters are listed in Table 6.

**Figure 13:**
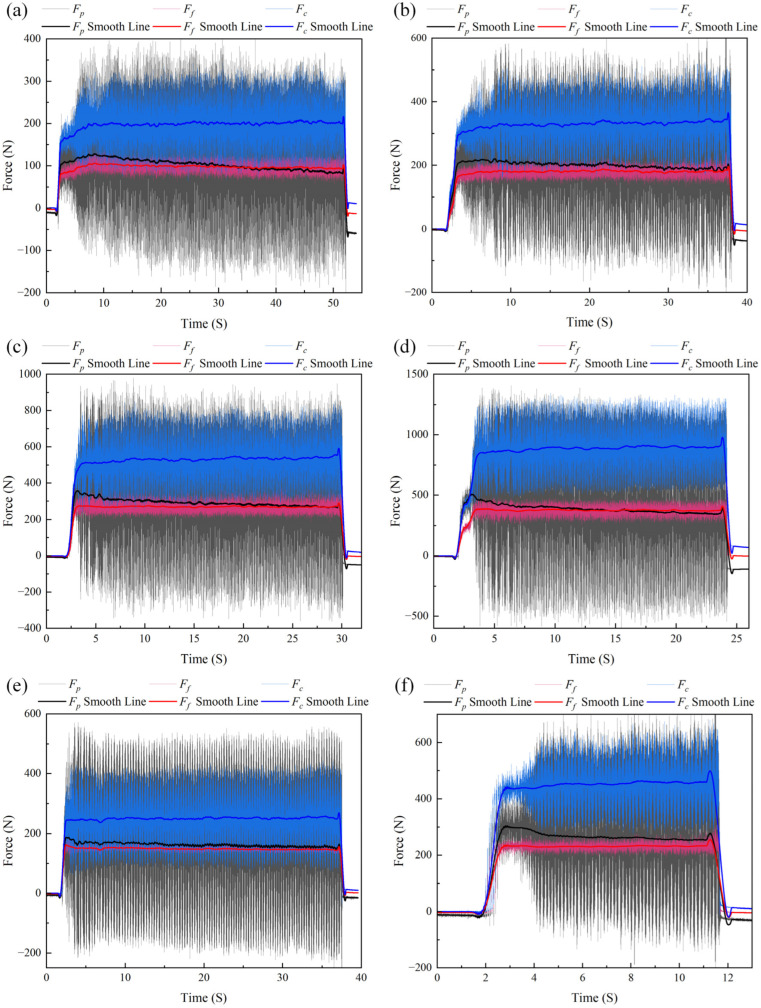
Cutting force measurement curves. (**a**) Test No. 1; (**b**) Test No. 2; (**c**) Test No. 3; (**d**) Test No. 4; (**e**) Test No. 5; and (**f**) Test No. 6.

Figure 13 shows the cutting force variation curves under four different sets of cutting parameters, including both the raw measurement data and the smoothed curves obtained after Savitzky–Golay filter fitting. A comparison of the original three-dimensional force curves shows that the noise level of Fp is significantly higher than that of Fc and Ff. This discrepancy primarily stems from the distinct noise characteristics arising from different signal synthesis principles of the three force components. According to the measurement principle described in Section 2.4, *F_p_* is obtained by arithmetic summation of the output signals from the four Silver Ink sensing elements. During the summation process, the noise from each channel is linearly superimposed and amplified, thereby introducing significant additive interference. In contrast, *F_c_* and *F_f_* are mainly derived from the differential operation of signals from two specific channels. Differential operation inherently suppresses common-mode noise shared by the two channels, thus providing inherent noise suppression. It is this fundamental difference in synthesis methodology that renders the *F_p_* channel more sensitive to the noise present in the system. Moreover, analyzing Test Nos. 1–4 reveals that while the cutting speed remains constant at 73 m/min, the three-component cutting forces all show a significant increasing trend as the feed rate and depth of cut are progressively raised from one test to the next. Secondly, a comparison between Test Nos. 5 and 1 shows that under the same feed rate (0.1 mm/r), a slight increase in the depth of cut coupled with a simultaneous rise in cutting speed results in only a modest increase in the three-component cutting forces. This indicates that when the feed rate remains unchanged, the mild softening effect induced by the higher cutting speed partially offsets the force increase resulting from the larger depth of cut. A further comparison between Test No. 2 and Test No. 6 shows that under the same depth of cut (0.8 mm), when both the feed rate and cutting speed are substantially increased, the three-component cutting forces rise significantly.

The observed trends align with the theoretical principles of metal cutting, demonstrating that the employed force sensor can effectively capture the influence of different cutting parameters on cutting forces during actual machining. This validates its reliable measurement capability throughout dynamic cutting processes.

**Figure 14:**
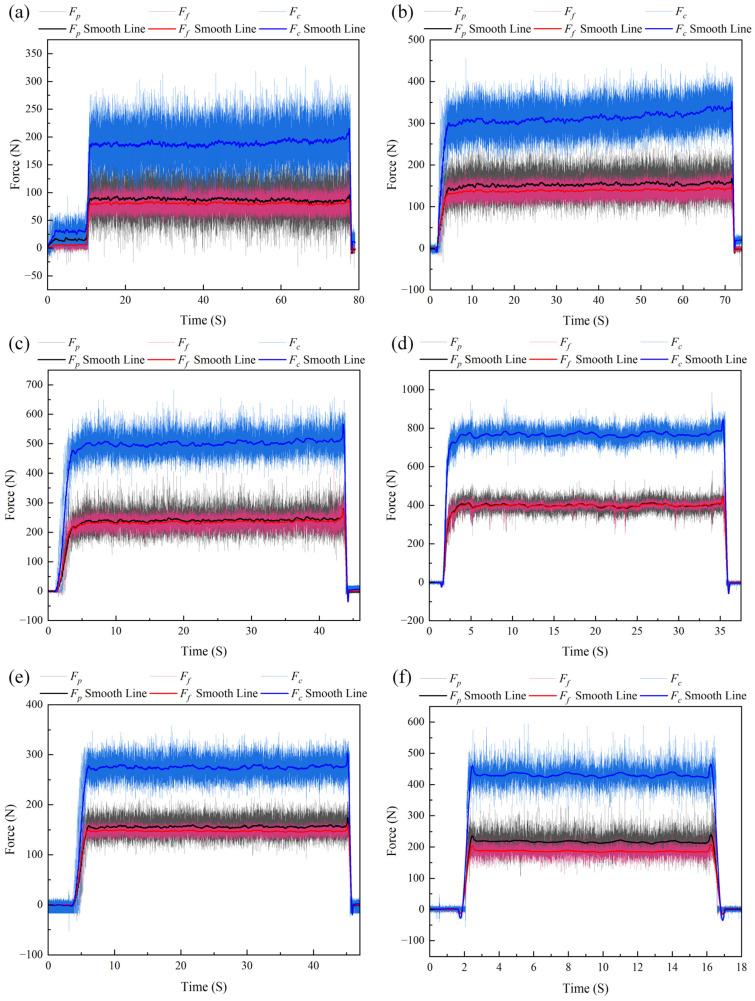
Kistler cutting force measurement curves. (**a**) Test No. 1; (**b**) Test No. 2; (**c**) Test No. 3; (**d**) Test No. 4; (**e**) Test No. 5; and (**f**) Test No. 6.

To quantitatively evaluate the accuracy and reliability of the developed PVDF piezoelectric film force sensor in dynamic cutting force measurement, a comparative cutting test was conducted under identical operating conditions using a Kistler 9272A dynamometer (Kistler Group, Winterthur, Switzerland) as the reference standard, as shown in Figure 12b. The resulting cutting force variation curves are presented in Figure 14.

**Figure 15:**
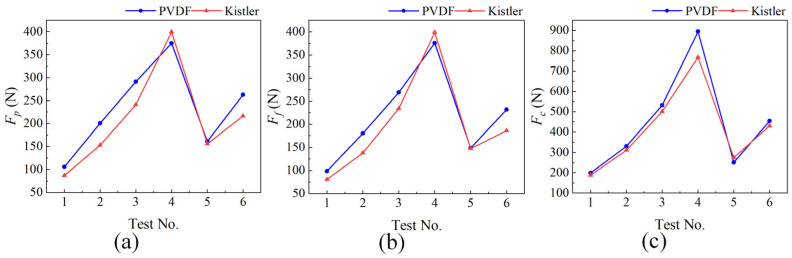
Comparison of three-dimensional cutting force measurements between the PVDF piezoelectric film force sensor and the commercial dynamometer. (**a**) Comparison of *F_p_*; (**b**) Comparison of *F_f_*; and (**c**) Comparison of *F_c_*.

To validate the measurement performance, the cutting force data corresponding to the stable cutting stage were selected for comparative analysis between the two sensors. The comparison curves of the three-dimensional cutting forces are shown in Figure 15. It can be observed that the three-dimensional cutting forces measured by the proposed PVDF sensor exhibit a high degree of consistency with those measured by the commercial dynamometer in terms of overall trend. However, certain quantitative deviations still exist. This deviation primarily arises from two aspects. The primary cause is the fundamental difference in the measurement coordinate systems between the two devices. The PVDF sensor is based on the tool mounting coordinate system, with its measurement reference point located at the tool tip, whereas the Kistler dynamometer has its measurement reference point at the center of its body and is installed beneath the tool holder. The inherent discrepancies in the spatial force transmission path, moment arm length, and structural stiffness distribution between the two systems result in inevitable differences in the absolute force values. The secondary cause is that although the least squares-based linear decoupling model adopted in this study effectively compensates for the dominant linear couplings in the system, residual nonlinear coupling errors have not been completely eliminated.

## 4. Conclusions

To overcome the challenges of contact interface force field nonlinearity in conventional PVDF piezoelectric film force sensors and the inability of a monolithic PVDF film to measure multi-directional forces, this paper proposes a novel solution: a PVDF piezoelectric film three-dimensional force sensor based on interface force field reconstruction and surface domain segmentation. The key conclusions drawn from this study are as follows:

(1) Based on the structural design of the uniform-load double-bossed elastic force-transmitting diaphragm, the contact interface force field of the PVDF piezoelectric film has been reconstructed and optimized, thereby enhancing the consistency and reliability of the sensor’s measurements.
(2) Based on the load-bearing surface domain segmentation technique, a PVDF piezoelectric film sensitive element group was constructed. Three-dimensional force measurement was achieved through the synergistic design of implementing a pre-stressed assembly structure and extracting signals from the sensitive element group.
(3) Quasi-static calibration test results show that the triaxial charge sensitivities of the force sensor are 52.63 pC/N, 55.96 pC/N, and 9.02 pC/N, respectively, with a linearity better than 4.6%. Dynamic calibration test results reveal that the natural frequency of the force measurement module is 4675.5 Hz.
(4) Dynamic cutting tests were conducted, which verified the sensor’s capability to capture dynamic triaxial cutting forces.

Future work will focus on an in-depth investigation of the mechanical coupling mechanisms in each direction, employing algorithms such as nonlinear decoupling to reduce interference errors in the Z-axis.

## Figures and Tables

**Table 1:** Performance parameters of the PVDF piezoelectric film.

Material	Film Thickness (μm)	Polarization Orientation	Piezoelectric Strain Constant (pC/N)	Relative Permittivity
PVDF	110	*d*_33_	*d*_33_ = 33	12

**Table 2:** Material properties of sensor components.

Name	Materials	Density (kg/m^3^)	Young’s Modulus (GPa)	Poisson’s Ratio
Housing	17-4ph	7750	196	0.27
Insulating layer	PI	1430	4.1	0.2
Piezoelectric film	PVDF	1780	2.96	0.35

**Table 3:** Comparison of key mechanical indicators under the two sensor structures.

Evaluation Indicator	Circular Flat Diaphragm	Uniform-Load Diaphragm
Maximum equivalent stress of PVDF	9.236 × 10^6^ Pa	2.7509 × 10^6^ Pa
Minimum equivalent stress of PVDF	1.6568 × 10^5^ Pa	2.2328 × 10^6^ Pa
Average equivalent stress of PVDF	1.128 × 10^6^ Pa	2.4317 × 10^6^ Pa
Stress concentration factor of PVDF	8.19	1.13
Maximum deformation of housing	2.7275 × 10^−4^ mm	9.185 × 10^−5^ mm
Dimensionless displacement ratio	1.36 × 10^−4^	4.59 × 10^−5^

**Table 4:** The main technical specifications of the sensor.

Force Component	Charge Sensitivity (pC/N)	Linearity	Cross-Interference Error		
			*F* *_x_*	*F* *_y_*	*F_z_*
*F* *_x_*	52.63	2.84%	-	1.77%	49.55%
*F* *_y_*	55.96	4.60%	5.64%	-	70.18%
*F_z_*	9.02	1.82%	0.34%	8.23%	-

**Table 5:** Cross-axis interference errors of the force sensor after decoupling.

Force Component	Cross-Interference Error		
	*F* *_x_*	*F* *_y_*	*F_z_*
*F* *_x_*	-	1.66%	17.49%
*F* *_y_*	0.01%	-	28.69%
*F_z_*	0.74%	0.77%	-

**Table 6:** Cutting test parameter settings.

Test No.	Cutting Speed (m/min)	Feed Rate (mm/r)	Cutting Depth (mm)
1	73	0.1	0.6
2	73	0.14	0.8
3	73	0.18	1.0
4	73	0.22	1.2
5	116	0.1	0.8
6	183	0.22	0.8

## Data Availability

The original contributions presented in this study are included in the article. Further inquiries can be directed to the corresponding author.
